# Estimated Number of Cases of High-Grade Cervical Lesions Diagnosed Among Women — United States, 2008 and 2016

**DOI:** 10.15585/mmwr.mm6815a1

**Published:** 2019-04-19

**Authors:** Nancy M. McClung, Julia W. Gargano, Ina U. Park, Erin Whitney, Nasreen Abdullah, Sara Ehlers, Nancy M. Bennett, Mary Scahill, Linda M. Niccolai, Monica Brackney, Marie R. Griffin, Manideepthi Pemmaraju, Troy D. Querec, Angela A. Cleveland, Elizabeth R. Unger, Lauri E. Markowitz, HPV-IMPACT Working Group, HPV-IMPACT Working Group, Sheelah Blankenship, Stephanie Allen, James Meek, Kyle Higgins, James Hadler, Lynn Sosa, Kayla Saadeh, Deana Fink, Michael Silverberg, Melissa E. Powell, Shannon Q. Allain, Christina Felsen, RaeAnne Bogart, Marina Oktapodas Feiler, Rebecca M. Dahl

**Affiliations:** ^1^Epidemic Intelligence Service, CDC; ^2^Division of Viral Diseases, National Center for Immunization and Respiratory Diseases, CDC; ^3^Department of Family and Community Medicine, School of Medicine, University of California at San Francisco; ^4^California Emerging Infections Program, Oakland, California; ^5^Oregon Health Authority, Public Health Division, Portland, Oregon; ^6^University of Rochester School of Medicine and Dentistry, Rochester, New York; ^7^Yale School of Public Health, New Haven, Connecticut; ^8^Vanderbilt University Medical Center, Nashville, Tennessee; ^9^Division of High-Consequence Pathogens and Pathology, National Center for Emerging and Zoonotic Infectious Diseases, CDC.; Vanderbilt University Medical Center; Vanderbilt University Medical Center; Yale School of Public Health; Yale School of Public Health; Yale School of Public Health; Connecticut Department of Public Health; California Emerging Infections Program; California Emerging Infections Program; Kaiser Permanente Northern California; Oregon Health Authority; Oregon Health Authority; University of Rochester School of Medicine and Dentistry; University of Rochester School of Medicine and Dentistry; University of Rochester School of Medicine and Dentistry; Maximus Federal.

Human papillomavirus (HPV) causes approximately 30,000 cancers in the United States annually (*1*). HPV vaccination was introduced in 2006 to prevent HPV-associated cancers and diseases (*1*). Cervical cancer is the most common HPV-associated cancer in women (*1*). Whereas HPV-associated cancers typically take decades to develop, screen-detected high-grade cervical lesions (cervical intraepithelial neoplasia grades 2 [CIN2], 3 [CIN3], and adenocarcinoma in situ, collectively CIN2+) develop within a few years after infection and have been used to monitor HPV vaccine impact (*1*–*3*). CDC analyzed data from the Human Papillomavirus Vaccine Impact Monitoring Project (HPV-IMPACT), a population-based CIN2+ surveillance system, to describe rates of CIN2+ among women aged ≥18 years during 2008–2016. Age-specific rates were applied to U.S. population data to estimate the total number of CIN2+ cases diagnosed in the United States in 2008* and in 2016. From 2008 to 2016, the rate of CIN2+ per 100,000 women declined significantly in women aged 18–19 years and 20–24 years and increased significantly in women aged 40–64 years. In the United States in 2008, an estimated 216,000 (95% confidence interval [CI] = 194,000–241,000) CIN2+ cases were diagnosed, 55% of which were in women aged 18–29 years; in 2016, an estimated 196,000 (95% CI = 176,000–221,000) CIN2+ cases were diagnosed, 36% of which were in women aged 18–29 years. During 2008 and 2016, an estimated 76% of CIN2+ cases were attributable to HPV types targeted by the vaccine currently used in the United States. These estimates of CIN2+ cases likely reflect changes in CIN2+ detection resulting from updated cervical cancer screening and management recommendations, as well as primary prevention through HPV vaccination. Increasing coverage of HPV vaccination in females at the routine age of 11 or 12 years and catch-up vaccination through age 26 years will contribute to further reduction in cervical precancers.

In 2006, HPV vaccine was licensed and recommended for routine vaccination in females aged 11 or 12 years and for catch-up vaccination through age 26 years (*1*). Two vaccines primarily have been used in the United States: until 2015, the quadrivalent vaccine, which in addition to HPV 6 and 11, targets high-risk, or oncogenic, HPV 16 and 18, and since 2016, 9-valent vaccine, which also targets high-risk HPV types 31, 33, 45, 52, and 58. HPV vaccination coverage among females aged 13–17 years has increased since 2007. In 2016, coverage of ≥1 dose was 65.1% and 3 doses was 43.0% (*1*).

The HPV-IMPACT sites are located in five surveillance network locations. The specific catchment areas, defined by county or zip code, were selected to provide a diverse population of women and a feasible population size and geographic area for complete case ascertainment; in total, approximately 1.5 million women reside in the catchment areas.^†^ HPV-IMPACT uses active surveillance of diagnostic pathology laboratories to collect all CIN2+ cases (*3*). Site staff members routinely audit all laboratories and gynecology practices serving catchment areas to ensure complete case ascertainment. Archived diagnostic specimens for type-specific HPV DNA detection of 37 types are obtained for cases in women aged 18–39 years (*2*). Age-stratified CIN2+ incidence rates per 100,000 women were calculated for each year (2008–2016)^§^; trends were evaluated using joinpoint models in Joinpoint software (version 4.6.0.0; National Cancer Institute) and reported as average annual percentage change (AAPC) with 95% CIs.^¶^

To estimate the number of CIN2+ cases in 2008 and 2016 by age group, the observed age-specific CIN2+ rates were applied to age-specific, annual U.S. population estimates.** HPV types were categorized as HPV16/18, HPV31/33/45/52/58, and other type/HPV-negative. To estimate the number of HPV type–specific cases, the age-specific HPV type distribution observed from typing data was applied to age-specific total CIN2+ estimates.^††^ Case estimates were rounded to the nearest 1,000 cases.^§§^ An analysis using higher and lower CIN2+ rates observed in specific HPV-IMPACT sites was performed to describe potential uncertainty in estimates.^¶¶^

During 2008–2016, a total of 23,489 CIN2+ cases were reported to HPV-IMPACT, and HPV DNA typing was performed for 11,581 of 16,590 (69.8%) cases in women aged 18–39 years. In 2008, HPV-IMPACT CIN2+ rates were highest in women aged 20–24 years (559 per 100,000 women [95% CI = 521–600]) and were lower in successively older age groups (Table). In 2016, CIN2+ rates were highest in women aged 25–29 years (480 [95% CI = 448–515]) and lower in each successively older age group. From 2008 to 2016, the rate of CIN2+ per 100,000 women declined significantly in women aged 18–19 years and 20–24 years and increased significantly in women aged 40–64 years.

**TABLE:** Age group–specific annual CIN2+ cases per 100,000 women, and average annual percentage change (AAPC)* — Human Papillomavirus Vaccine Impact Monitoring Project, United States, 2008–2016

Age group (yrs)^†^	CIN2+ rate (95% CI)									AAPC* (95% CI)
	Year of diagnosis									
	2008	2009	2010	2011	2012	2013	2014	2015	2016	
18–19	206	118	83	27	20	15	9	8	12	–38.5
	(172 to 248)	(93 to 151)	(62 to 110)	(16 to 45)	(11 to 36)	(7 to 29)	(4 to 23)	(3 to 20)	(5 to 26)	(–44.6 to –31.8)
20–24	559	499	412	381	351	271	191	185	151	–14.9
	(521 to 600)	(463 to 537)	(380 to 447)	(350 to 415)	(322 to 383)	(246 to 300)	(169 to 215)	(163 to 209)	(132 to 173)	(–17.1 to –12.6)
25–29	504	506	499	466	461	495	442	427	480	–1.4
	(469 to 542)	(471 to 544)	(464 to 536)	(433 to 502)	(428 to 497)	(461 to 531)	(411 to 476)	(397 to 460)	(448 to 515)	(–2.8 to 0.1)
30–34	371	363	334	363	337	366	398	420	419	2.1^§^
	(339 to 406)	(332 to 397)	(304 to 366)	(333 to 396)	(308 to 368)	(336 to 398)	(367 to 431)	(389 to 454)	(388 to 453)	(–0.4 to 4.8)
35–39	202	235	238	226	213	229	210	276	276	2.7
	(179 to 228)	(210 to 263)	(213 to 267)	(202 to 254)	(189 to 240)	(205 to 257)	(187 to 236)	(250 to 306)	(250 to 306)	(–0.1 to 5.6)
40–44	143	147	166	154	149	172	172	171	175	2.4
	(124 to 165)	(127 to 169)	(145 to 190)	(134 to 177)	(129 to 171)	(150 to 196)	(151 to 196)	(149 to 195)	(153 to 200)	(0.9 to 3.9)
45–49	87	88	73	95	101	92	112	92	112	3.4
	(72 to 104)	(74 to 105)	(60 to 89)	(80 to 113)	(86 to 120)	(77 to 110)	(95 to 132)	(77 to 110)	(95 to 132)	(0.3 to 6.6)
50–54	54	51	51	53	48	67	77	76	65	5.5
	(42 to 68)	(40 to 64)	(40 to 64)	(42 to 66)	(38 to 62)	(54 to 82)	(63 to 93)	(63 to 92)	(53 to 80)	(1.6 to 9.6)
55–59	30	36	45	41	38	43	44	41	58	5.3
	(22 to 41)	(27 to 49)	(34 to 58)	(31 to 53)	(29 to 50)	(33 to 56)	(34 to 57)	(32 to 54)	(46 to 72)	(1.4 to 9.2)
60–64	30	24	26	41	41	33	32	42	48	6.1
	(20 to 43)	(16 to 36)	(18 to 38)	(31 to 55)	(31 to 55)	(24 to 45)	(23 to 44)	(32 to 55)	(37 to 62)	(0.7 to 11.9)
≥65	14	13	14	11	13	12	10	13	12	–1.6
	(10 to 19)	(10 to 18)	(10 to 19)	(8 to 16)	(9 to 18)	(9 to 17)	(7 to 14)	(10 to 18)	(9 to 17)	(–4.7 to 1.6)

**Abbreviations:** CI = confidence interval; CIN2+ = cervical intraepithelial neoplasia grades 2, 3, and adenocarcinoma in situ.

* Trends were measured with AAPC in age-stratified rates, and were considered to increase (AAPC>0) or decrease (AAPC<0) if the 95% confidence interval did not include 0; otherwise, trends were considered stable.

^†^ The median age at CIN2+ diagnosis was 28 years (interquartile range [IQR] = 24–35 years) in 2008 and 32 years (IQR 27–39 years) in 2016.

^§^ In women aged 30–34 years, a joinpoint was detected. From 2008 to 2012, the annual percentage change (APC) indicated that rates were stable (−1.4 [95% CI = −6.5 to 4.1]), but from 2012 to 2016, the APC indicated that rates were increasing (5.8 [95% CI = 0.7 to 11.1]).

Extrapolating age-specific HPV-IMPACT rates to the U.S. population, an estimated 216,000 (95% CI = 194,000–241,000) CIN2+ cases were diagnosed in the United States in 2008 (Figure 1), including 119,000 (55%) in women aged 18–29 years, 57,000 (26%) in women aged 30–39 years, and 40,000 (18%) in women aged ≥40 years. Among the estimated 216,000 cases, 165,000 (76%) were attributable to 9-valent vaccine types (111,000 [52%] to HPV16/18 and 54,000 [25%] to HPV31/33/45/52/58) (Figure 2). Among women aged 18–24 years, 52% of CIN2+ cases were HPV16/18-attributable. Of the 165,000 CIN2+ cases attributable to 9-valent vaccine types, 91,000 (55%), 43,000 (26%), and 31,000 (19%) occurred in women aged 18–29, 30–39, and ≥40 years, respectively.

**FIGURE 1:**
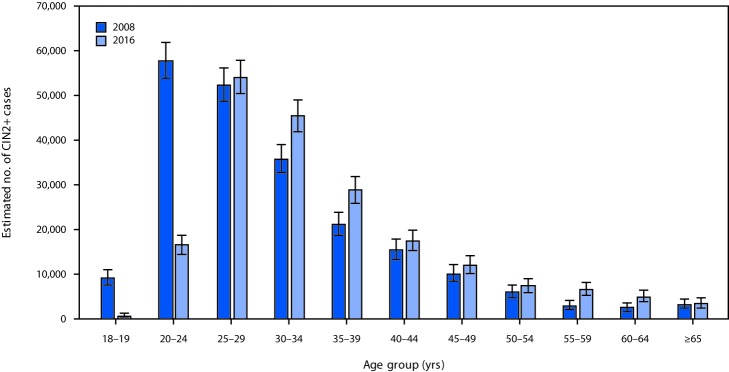
Estimated number of diagnosed CIN2+ cases,* by age group — United States, 2008 and 2016 **Abbreviation**: CIN2+ = cervical intraepithelial neoplasia grades 2, 3, and adenocarcinoma in situ. * Error bars indicate 95% confidence intervals, which were calculated by applying the upper and lower limits of CIN2+ rates to the age-specific U.S. population.

**FIGURE 2:**
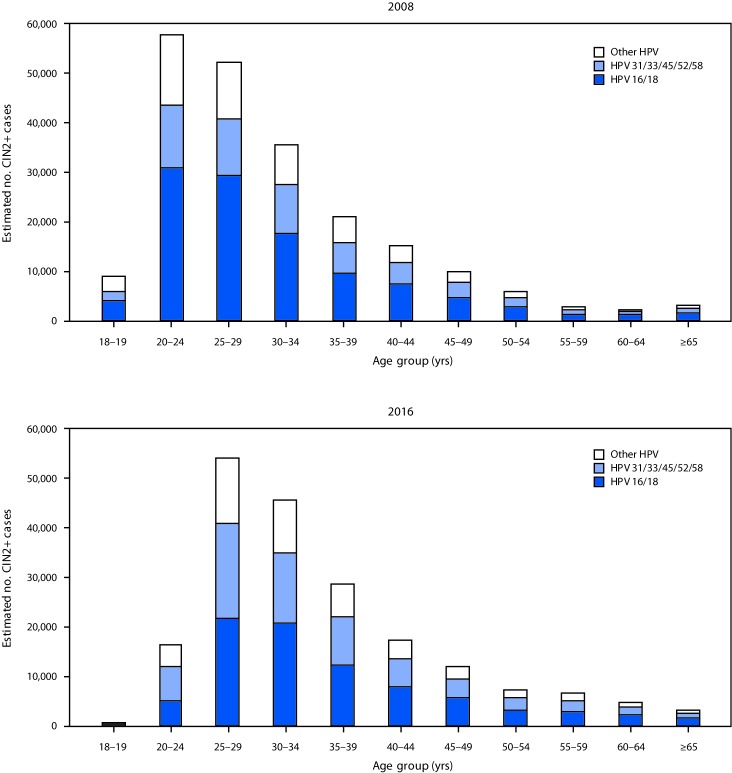
Estimated number of diagnosed CIN2+ cases, by human papillomavirus (HPV) type* and age group — United States, 2008 and 2016 **Abbreviation:** CIN2+ = cervical intraepithelial neoplasia grades 2, 3 and adenocarcinoma in situ. * Type-specificity for 2008 was based on typing data from 2008, and for 2016, was based on typing data from 2015 (most recently available) applied to 2016 case counts by diagnosis grade. HPV type group “other HPV” includes HPV-negative cases.

In 2016, an estimated 196,000 (95% CI = 176,000─221,000) CIN2+ cases were diagnosed in the United States, including 71,000 (36%) in women aged 18–29 years, 74,000 (38%) in women aged 30–39 years and 51,000 (26%) in women aged ≥40 years (Figure 1). Among the 196,000 total cases, 150,000 (76%) were attributable to 9-valent vaccine types, including 84,000 (43%) to HPV16/18 and 66,000 (34%) to HPV31/33/45/52/58 (Figure 2). Among women aged 18–24 years, 30% of CIN2+ cases were HPV 16/18-attributable. Of the 150,000 CIN2+ cases attributable to 9-valent vaccine types, 53,000 (35%), 57,000 (38%), and 40,000 (27%) occurred in women aged 18–29, 30–39, and ≥40 years, respectively.

## Discussion

This report describes the first estimates of the number of U.S. CIN2+ cases developed from population-based data. In 2008 and 2016, an estimated 216,000 and 196,000 CIN2+ cases were diagnosed, respectively; in both years, 76% were attributable to 9-valent HPV vaccine types. A previous U.S. estimate of 177,469 CIN2+ cases in 2000 was limited by extrapolation from health claims data among privately insured women (*4*). To estimate U.S. CIN2+ cases, this report also extends previously reported HPV-IMPACT CIN2+ rates, by including rates in women aged ≥40 years (*3*). Two additional population-based surveillance systems have published CIN2 or CIN3 rates, but have not used them to estimate numbers of U.S. CIN2+ cases (*5*,*6*). Rates from those systems were not incorporated into estimates presented in this report because those rates were calculated using a denominator of screened women, did not include HPV typing data, or did not include data on all age groups and years.

Both the estimated number and rates of U.S. CIN2+ cases in this report must be interpreted in the context of cervical cancer prevention strategies, including HPV vaccination and cervical cancer screening. CIN2+ is detected through cervical cancer screening and referral for diagnostic biopsy; thus, changes in screening and management recommendations that occurred during the surveillance period in this report affect CIN2+ detection (*7*,*8*). In 2008, the recommended age for initiation of screening was within 3 years of initiation of sexual activity or by age 21 years, with annual screening thereafter recommended by many professional organizations. By 2016, the recommended age for screening initiation was 21 years, and screening intervals had increased to every 3 years with cytology alone, or every 5 years with cytology plus HPV testing in women aged ≥30 years. Older age at screening initiation, longer screening intervals, and more conservative management in young women might be expected to reduce the number of CIN2+ cases detected in younger age groups in whom lesions are most likely to regress and shift detection of some CIN2+ to older age groups, resulting in a transient increase in rates (*3*,*5*). In younger age groups, the decline in HPV 16/18-attributable CIN2+, targeted by the quadrivalent vaccine from 2006 to 2015, also reflects the impact of the U.S. HPV vaccination program. Some of the increases in older age groups could be attributable to use of HPV testing, which is more sensitive than cytology, as part of cervical cancer screening, as has been predicted by modeling studies (*9*).

The findings in this report are subject to at least three limitations. First, U.S. CIN2+ cases were extrapolated from population-based surveillance in five communities, which was not designed to be nationally representative. Compared with the U.S. population, HPV-IMPACT catchment areas have a similar proportion of white women, a slightly higher proportion of black and Asian women, and a lower proportion of Hispanic women. Age-stratified rates were used to project to the U.S. population; however, this analysis did not adjust for race or other population characteristics, such as screening practices, that could affect the estimates. If actual U.S. CIN2+ rates are higher or lower than HPV-IMPACT CIN2+ rates, U.S. case numbers could be incorrectly estimated. Second, HPV type distribution in age groups ≥40 years was based on the distribution in women aged 30–39 years; prior HPV typing data in older age groups suggest that these calculations might overestimate contributions of 9-valent vaccine types in women aged ≥40 years (*10*). Finally, this analysis could not fully differentiate the factors influencing changes in CIN2+ development and detection, including screening and management recommendations and vaccination. However, previous studies have demonstrated that declining CIN2+ rates are not fully explained by changes in screening (*3*), and the proportion of CIN2+ attributable to vaccine types is declining (*2*).

This first estimate of the number of U.S. CIN2+ cases derived from population-based data, including the percentage that could be prevented by vaccination, is important for understanding CIN2+ trends across all age groups and will help to better identify the impact of both vaccination and changes to cervical screening and management guidelines. Increasing coverage of HPV vaccination in females at the routine age of 11 or 12 years and catch-up vaccination through age 26 years for those not adequately vaccinated previously will contribute to further reduction in cervical precancers.

**Summary**

**What is already known about this topic?**

Cervical cancer is the most common human papillomavirus (HPV)-associated cancer in women, and high-grade cervical lesions (CIN2+) have been used to monitor HPV vaccine impact.

**What is added by this report?**

During 2008–2016, CIN2+ rates in a population-based surveillance system declined in women aged 18–24 years. The estimated numbers of U.S. CIN2+ cases were 216,000 (2008) and 196,000 (2016), with an estimated 76% attributable to 9-valent HPV vaccine types.

**What are the implications for public health practice?**

Cervical cancer prevention strategies include both HPV vaccination and screening. The reduction in CIN2+ attributable to vaccine types in young women demonstrates impact of the HPV vaccination program. Continued efforts to increase coverage and encourage vaccination at the routine ages (11–12 years) can increase vaccine impact on cervical disease in the United States.

## Footnotes

* Surveillance through HPV-IMPACT began in 2008. Although the vaccination program began in 2006, because of the time it takes for cervical precancers to develop and be detected through screening, rates in 2008 should not be affected by vaccination and should therefore represent prevaccine era rates.

† The HPV-IMPACT sites are located in five of the 10 Emerging Infections Programs (EIP) surveillance network locations (https://www.cdc.gov/ncezid/dpei/eip/eip-sites.html); the specific catchment areas within the EIP sites are New Haven County, Connecticut; Monroe County, New York; Davidson County, Tennessee; portions of Alameda County, California; and portions of Washington and Multnomah Counties, Oregon. (https://www.cdc.gov/ncird/surveillance/hpvimpact/index.html).

§ Population denominators were based on county-level data from CDC’s National Center for Health Statistics (https://www.cdc.gov/nchs/nvss/bridged_race/data_documentation.htm); for California and Oregon sites, county estimates were adjusted for the specific catchment area using American Community Survey data.

¶ Trends were measured with AAPC in age-stratified rates and were considered to increase (AAPC>0) or decrease (AAPC<0) if the 95% CI did not include 0; otherwise trends were considered stable.

** The total number of U.S. CIN2+ cases in each age group and 95% CIs were estimated by multiplying the age-specific CIN2+ rates and upper and lower CIs by the age-specific U.S. population estimates. For example, in women aged 20–24 years, the observed HPV-IMPACT CIN2+ rate in 2008 was 559 (95% CI = 521–600) per 100,000 women, and there were 10,339,566 women aged 20–24 years in the United States; therefore, the estimated number of U.S. CIN2+ cases in this age group was 559 x 10,339,566/100,000 = 58,000 CIN2+ cases (rounded to the nearest 1,000). The total cases were estimated by summing the age-specific estimates.

†† HPV typing data were available for approximately 70% of cases in women aged 18–39 years, and the proportion positive for each HPV type group varied by lesion grade. Therefore, to estimate the number of cases in each age group attributable to each HPV type group, the proportion positive for each HPV type group within each lesion grade was multiplied by the number of cases having each lesion grade within each age group. For age groups <40 years, the observed HPV type proportion within each lesion grade for each age group was applied. For age groups ≥40 years, the observed HPV type proportion within each lesion grade for cases among women aged 30–39 years was applied.

§§ Components might not sum to totals because of rounding.

¶¶ A range of lower and higher national estimates were developed based on data from two sites with historically lower CIN2+ rates (California and Tennessee) and from two sites with historically higher CIN2+ rates (Connecticut and New York). The low and high estimates of the number of prevaccine era CIN2+ cases were 159,000 (95% CI = 131,000–197,000) and 283,000 (95% CI = 246,000–328,000); the low and high estimates of the number of 2016 CIN2+ cases were 179,000 (95% CI = 150,000–217,000) and 210,000 (95% CI = 177,000–253,000).
